# Dynamics of American tegumentary leishmaniasis in a highly endemic region for *Leishmania (Viannia) braziliensis* infection in northeast Brazil

**DOI:** 10.1371/journal.pntd.0006015

**Published:** 2017-11-02

**Authors:** Juliana Silva, Adriano Queiroz, Izabella Moura, Rosana S. Sousa, Luiz Henrique Guimarães, Paulo Roberto Lima Machado, Marcus Lessa, Ednaldo Lago, Mary E. Wilson, Albert Schriefer

**Affiliations:** 1 Serviço de Imunologia, Hospital Universitário Professor Edgard Santos, Universidade Federal da Bahia, Salvador, Bahia, Brazil; 2 Instituto Nacional de Ciência e Tecnologia–Doenças Tropicais (INCT-DT), Brazil; 3 Departments of Internal Medicine and Microbiology, University of Iowa and the VA Medical Center, Iowa City, IA, United States of America; 4 Departamento de Ciências da Biointeração, Instituto de Ciências da Saúde, Universidade Federal da Bahia, Salvador, Bahia, Brazil; National Institutes of Health, UNITED STATES

## Abstract

**Background:**

American Tegumentary Leishmaniasis (ATL) caused by *Leishmania braziliensis* is endemic in Corte de Pedra, Northeast Brazil. Most *L*. *braziliensis* infections manifest as localized cutaneous leishmaniasis (CL). Disseminated manifestations include mucosal leishmaniasis (ML), present at a low constant level for several decades, and newly emerging disseminated leishmaniasis (DL). Surprisingly, DL has recently surpassed ML in its spatial distribution. This led us to hypothesize that distinct forms of ATL might spread in different patterns through affected regions.

**Methodology/Principal findings:**

We explored the incidence and geographic dispersion of the three clinical types of ATL over a span of nearly two decades in Corte de Pedra. We obtained the geographic coordinates of the homes of patients with ATL during 1992–1996, 1999–2003 and 2008–2011. The progressive dispersion of ML or DL in each time period was compared to that of CL in 2008–2011 with the Cusick and Edward’s geostatistical test. To evaluate whether ATL occurred as clusters, we compared each new case in 2008–2011 with the frequency of and distance from cases in the previous 3 to 12 months. The study revealed that DL, ML and CL actively spread within that region, but in distinct patterns. Whereas CL and DL propagated in clusters, ML occurred as sporadic cases. DL had a wider distribution than ML until 2003, but by 2011 both forms were distributed equally in Corte de Pedra. The incidence of ML fluctuated over time at a rate that was distinct from those of CL and DL.

**Conclusions/Significance:**

These findings suggest that CL and DL maintain endemic levels through successive outbreaks of cases. The sporadic pattern of ML cases may reflect the long and variable latency before infected patients develop clinically detectable mucosal involvement. Intimate knowledge of the geographic distribution of leishmaniasis and how it propagates within foci of active transmission may guide approaches to disease control.

## Author summary

American tegumentary leishmaniasis (ATL) caused by *Leishmania braziliensis* is characterized by lesions to the skin and/or mucosal surfaces of the oropharynx. It is widely distributed in endemic regions of northeast Brazil and has been difficult to control. Three common clinical forms of *L*. *braziliensis* infections are localized skin ulcers called cutaneous leishmaniasis (CL), mucosal leishmaniasis (ML) affecting mucosal surfaces, and disseminated leishmaniasis (DL), a recently described form with widespread skin lesions. Using GPS and epidemiologic data we explored the incidence and pattern of spread of ATL in the highly endemic region of Corte de Pedra, Brazil between 1992 and 2011. Geographic clusters of CL and DL cases were observed. In contrast, there was a sporadic non-clustered pattern of ML cases in the study area. The numbers of new cases of CL and DL presented similar fluctuation during the study period, but ML incidences were never correlated to those of CL and DL. We conclude that all forms of ATL actively spread within affected foci, but in different patterns. CL and DL cases occur in clusters suggesting active spread of causative parasite strains, whereas ML cases occurred in a sporadic pattern suggesting it may emerge due to factors such as host immunity or environmental conditions.

## Introduction

Leishmaniasis is a vector borne disease whose clinical presentations can be categorized in two broad groups: visceral and tegumentary leishmaniasis. The latter causes lesions in skin, and mucosal surfaces of the upper airway and digestive tracts [1]. Approximately 0.7 to 1.2 million new cases of tegumentary leishmaniasis occur every year worldwide [2].

American tegumentary leishmaniasis (ATL) extends from Mexico to Argentina [3], and is caused by various species of the *Leishmania braziliensis* and *Leishmania mexicana* complexes of parasites [4]. *Leishmania (*subgenus *Viannia) braziliensis* is responsible for the majority of ATL cases in South America, occurring at times in the form of localized cutaneous (CL), and mucosal leishmaniasis (ML) [5]. This species has also been implicated in the emergence of the new syndrome disseminated leishmaniasis (DL), in which patients most commonly present with approximately fifty skin lesions spread throughout different body parts, often with involvement of the oropharyngeal mucosa [6–9]. ML and DL are severe, hard to treat variants of ATL, which may result in disfiguring outcomes. These deserve particular attention during implementation of control measures.

Some epidemiologic reports have called attention to differences in the spatial organization of distinct forms of ATL. As examples, it has been shown that in Peru and Ecuador CL has a countrywide distribution, whereas ML is limited to the areas covered by the Amazon rain forest [10, 11]. We previously reported that ML and DL segregate differently within one of the foci with the highest endemic rates of *L*. *braziliensis* in northeast Brazil, in the region of Corte de Pedra [12]. We also observed significant clustering between new and recently diagnosed DL cases, indicating that proximity to a patient with DL is a risk factor for developing this form of leishmaniasis [12]. DL has emerged as the prominent form of disseminated disease caused by *L*. *braziliensis* in this region of Brazil.

Given our findings that distinct *L*. *braziliensis* clades are associated with specific forms of leishmaniasis [13], we reasoned that their patterns of spread over time may provide clues as to the mode of propagation of those parasite strains within the endemic population. In this study, we explored incidence and geographic localization of the three main clinical forms of ATL in patients presenting to a clinic in Corte de Pedra, over a period of nearly two decades. This data allowed us to evaluate the dynamics of ML and DL during the time intervals of 1992–1996, 1999–2003, and 2008–2011. These were compared to the baseline current data for CL, the most common form of the disease, which assumes a wide distribution within the region.

We report that the three forms of ATL actively spread within the affected focus, but in different patterns. CL and DL occur in clusters of cases, whereas ML occurs in a sporadic manner. A deeper understanding of the patterns of spatial spread may suggest the most efficacious venues to target in efforts to control the disease.

## Methods

### Study area

The Corte de Pedra region is composed of 20 municipalities in a rural area previously dominated by the Atlantic rain forest. The sand fly species *Lutzomyia (Nyssomyia) whitmani* and *Lu*. *(N*.*) intermedia*, which transmit *L*. *braziliensis*, are part of the local fauna. Residents in this area work mostly in agriculture, often close to primary or secondary forests. Among the population, there is little migration in or out of the region. The mean time of study participant residence at their addresses at the time of diagnosis and parasite sampling was 17 years (more precisely: mean = 17.6 years; median = 18.0 years; minimum time of residence = 1.0 year; maximum time of residence = 70.0 years). More than 90% of the study participants lived on farms.

### Study sample

Three distinct time-periods were considered in the study. Two were historical derived from subjects’ records, and the most recent included subjects actively enrolled according to the criteria of approximately two CL patients for each ML or DL subject, matched for month of diagnosis. Almost all ML and DL patients diagnosed in the region during this last period were included in the study. We then included roughly double the number of CL subjects relative to ML and DL to partially account for the fact that CL is much more frequent in Corte de Pedra, and thus avoid underrepresenting its actual geographic distribution in the region.

The historic samples consisted of 21 DL patients enrolled between 1992 and 1996, and 30 ML, 30 DL and 30 CL cases enrolled between 1999 and 2003. Geographic information was not available on any subjects other than DL individuals before 1999. The third, most recent sample consisted of 35 patients with ML, 76 with DL, and 225 with CL enrolled between 2008 and 2011. All cases in this study were self-referred to and diagnosed at the health post of Corte de Pedra. The health post services approximately 70% of the ATL patients in the region.

### Disease definitions

All subjects resided in the *L*. *braziliensis* endemic region. Clinical criteria for CL included fewer than 10 ulcerative skin lesions without evidence of mucosal involvement. DL was defined as a disease with more than 10, acneiform, inflammatory papular or ulcerative skin lesions spread over 2 or more body areas, with or without mucosal involvement. ML was defined by metastatic mucosal lesions affecting the nose, palate, pharynx, or larynx but not contiguous with primary cutaneous lesions, with or without the skin lesions of CL. Additionally, all patients had their diagnosis confirmed by at least two of the following criteria: (1) live parasites isolated in culture from lesion aspirates; (2) parasites visualized on lesion histopathology; and (3) delayed type hypersensitivity skin test to leishmania antigen (LST, Leishmania skin test). All subjects enrolled between 2008 and 2011 also had infection confirmed by parasite DNA detection in lesion biopsy specimens by PCR [14].

### Diagnostic and laboratory procedures

Leishmania cultures were prepared from aspirates of the borders of skin or mucosal lesions. Aspirate material was immediately suspended in biphasic LIT/NNN medium and incubated at 26°C for one to three weeks. The species of *Leishmania* promastigotes from positive cultures was confirmed by PCR as follows. Suspensions were transferred to Schneider’s medium with 10% heat-inactivated foetal calf serum and 2 mM L-glutamine, and incubated at 26°C until they reached a density of 10^7^ cells/mL. 1.7 x 10^7^ promastigotes were incubated in 150 μL of TELT buffer (Tris HCl 50 mM, EDTA 62.5 mM, LiCl 2.5 mM, Triton 100x 4%) for 5 min at room temperature. 150 μL of phenol-chloroform was added, and cells were vortexed and pelleted by microcentrifugation at 13,000 rpm for 5 min. DNA was ethanol precipitated, washed with 100% ethanol, air dried, re-suspended in 100 μL of Tris-EDTA (Tris-HCl 10 mM, EDTA 1 mM), and stored at -70°C. Aliquots of stored DNA were adjusted to 20 ng/μL before confirmation of *Leishmania* species was performed by PCR [14]. For detecting parasite DNA in patients’ skin and nasal mucosa, biopsy specimens from the lesions borders were stored in RNA Later solution (Ambion, Life Technologies, Thermo Fisher Scientific, USA) immediately after the procedure in the field, and kept at room temperature for approximately six hours until they were stored at 4°C in the laboratory. Two to three days later, nucleic acids were extracted from the skin and mucosal fragments using the DNA Purification kit (Promega Co., USA), according the manufacturer’s recommendations.

The leishmania skin test was performed with antigen prepared from a stock of *L*. *braziliensis* isolated from a localized cutaneous leishmaniasis patient of Corte de Pedra, maintained in our collection of frozen stocks of parasites. It has been used as our standard antigen for LST during diagnosis of ATL in the region. LST test was considered positive if a region of induration 5 mm or greater formed at the antigen injection site 48 to 72 hours post-test administration. Histopathology was considered positive when *Leishmania* spp. amastigotes were observed upon thorough examination of H&E stained biopsy fragments.

The above described diagnostic procedures were performed in all patients that participated the study, except for the detection of parasite DNA by PCR in biopsy specimens. PCR was only performed on samples of patients enrolled between 2008 and 2011.

### Mapping ATL patients in the study area

High-resolution distribution of ATL cases was determined by acquisition of geographic coordinates of likely places of disease transmission by global positioning system. Geographic coordinates were obtained using a Brunton Multi-Navigator GPS apparatus (Brunton Company, Riverton, WY, USA), which has a precision range of 15 m. Because leishmaniasis is believed to be transmitted mostly within plantations where most residents of the region live and work, patients’ residences were used as reference points for standardization purposes. The data were statistically evaluated as described below, and plotted for visual inspection onto a high-definition satellite photograph of Corte de Pedra region (ENGESAT, Curitiba, Brazil) using ArcGis version 10 software (Environmental Systems Research Institute Inc., Redlands, CA, USA).

### Statistical analyses

Comparisons between global distributions of different forms of ATL in Corte de Pedra employed the Cuzick and Edward's test (Clusterseer version 2.3, Terraseer Inc., Ann Arbor, MI, USA). To analyse whether proximity to a previous ATL case was accompanied by an increased frequency of ATL diagnosis among residents of the region, we used the ruler tool found in the ArcGIS software to measure the distances between the residence of each new case occurring between 2008 and 2011 (novel cases), and the homes of all cases occurring in the preceding 3, 6 or 12 months relative to the novel case (recent cases). The resulting data were stratified into discrete distance intervals of 0–2500, 2501–5000, 5001–7500, 7501–10000 and 10001–12500 meters from the novel cases. Then Spearman correlation between the number of closest recent cases and the distances from the novel cases was tested (Graphpad Prism version 5, Graphpad Software Inc. La Jolla, CA, USA). In these correlations, for each novel (i.e. newly diagnosed) case of leishmaniasis, the closest recent case of the same clinical type was determined. Then, the number of newly diagnosed cases that presented a closest recent case at a discrete 2.5 km distance interval was plotted against the distance interval units. The analysis was carried out for the entire set of ATL cases, and separately for each of the three forms of ATL (i.e. CL, DL and ML). For each novel case, only the closest recent case of the same form of disease was employed in the analysis, except when overall ATL was under consideration. Correlation between the incidence of ATL overall, and the incidence of CL, ML and DL separately, employed Pearson’s test (Graphpad Prism version 5, Graphpad Software Inc. La Jolla, CA, USA). Values of p<0.05 were considered significant.

### Ethics statement

The study was approved by the local Institutional Review Board (IRB) of the Federal University of Bahia (CAAE– 3041.0.000.054.07). Written consent was obtained from all subjects that participated in the study, including prospectively enrolled and historical subjects. The study was explained to and the written consent were obtained from all patients by the research team at the moments of their homes' geographic coordinates acquisition by GPS. Confidentiality was maintained by removing identification of study participants from their data records used in the research.

## Results

### ATL presents a long-term dynamic propagation within the affected region

The cumulative distribution of subjects with CL during the 2008–2011 period was the most widespread and stable collection of ATL patients in the dataset. Therefore, in order to track the spatial progression of DL and ML, we compared the distribution of DL and ML over time to the distribution of CL occurring in Corte de Pedra between 2008 and 2011. In these comparisons, the significances of the Cusick and Edward’s test were used as a metric of spread for each of the less common forms of ATL. The less significant the comparison to the cumulative distribution of 2008–2011 CL cases, the wider the spread of DL or ML.

**Fig 1:**
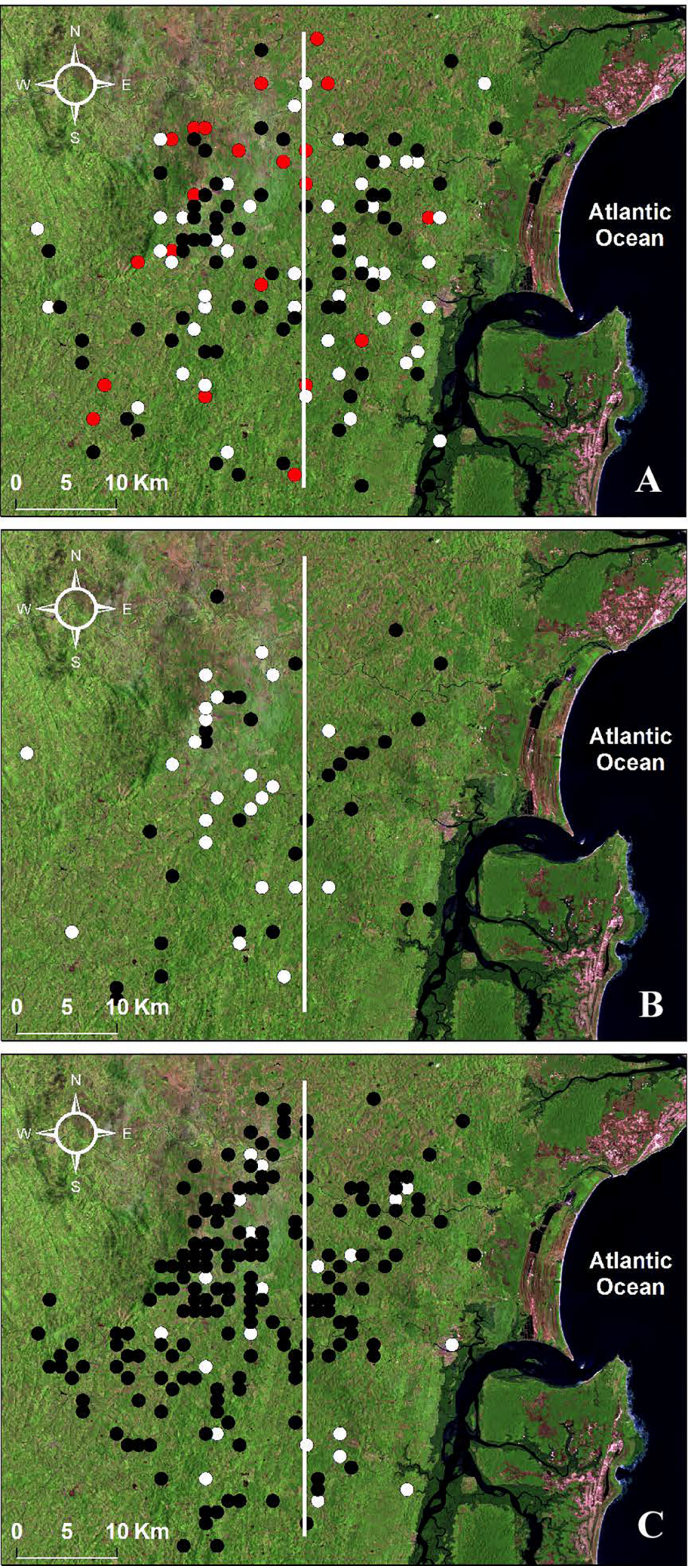
Dynamic distribution of ATL in Corte de Pedra. (A) Cumulative distribution of DL during three time periods: (red) 1992–1996, (white) 1999–2003, (black) 2008–2011. (B) Cumulative distribution of ML during two time periods: (white) 1999–2003, (black) 2008–2011. (C) Cumulative distribution of CL during two time periods: (white) 1999–2003, (black) 2008–2011.

The satellite views in Fig 1 depict Corte de Pedra divided by a line into inner and coastal halves of roughly similar areas to facilitate visual inspection. Fig 1A shows a progressive spread of DL in that region, with cases of the disease more often occurring within or very close to the inner half during the 1992–1996 period. The disease spreads toward the coastal half during the second (1999–2003) and third (2008–2011) study periods, during which DL cases assumed a more even distribution over Corte de Pedra. This visual impression was confirmed by Cusick and Edward's comparisons documenting significant difference between distributions of DL in 1992–1996 and CL in 2008–2012, but non-significant differences in 1996–2003 or 2008–2011 (Table 1). The increasing values of the comparisons significances through the three periods indicate the progressive nature of the spread of this disease in the region.

**Table 1:** Cuzick and Edwards test significance (p) values obtained from comparisons between distributions of localized cutaneous leishmaniasis (CL) cases diagnosed between 2008 and 2011, and disseminated (DL) or mucosal (ML) leishmaniais cases diagnosed during three discrete time periods: 1993 to 1996, 1999 to 2003, and 2008 to 2011.

ATL* form	Cuzick and Edwards’ p values		
	1993–1996	1999–2003	2008–2011
DL^#^	0.000034	0.078882	0.632225
ML^&^	Not applicable	0.000001	0.116288

* ATL: American tegumentary leishmaniasis.

^#^ DL: Disseminated leishmaniasis.

^&^ ML: Mucosal leishmaniasis.

Likewise, ML progressively acquired an even spatial spread throughout Corte de Pedra between 1999–2003 and 2008–2011 (Fig 1B). This was confirmed by a significantly different distribution when ML is compared to CL (2008–2011) in the earlier time, but a non-significant difference from CL in the latter period, according to Cusick and Edward`s test (Table 1).

Fig 1C shows the home locations of CL subjects enrolled between 2008 and 2011 that was used in the Cusick and Edward’s comparisons with DL and ML above. The figure demonstrates that CL was also evenly spread in Corte de Pedra during the 1999–2003 time period.

### CL and DL, but not ML occur as clusters of cases

Our prior reports demonstrated that distinct strains of *L*. *braziliensis* are associated with the different clinical types (DL, ML, CL) [13, 15]. Clustering of cases of similar clinical type would suggest that vector borne transmission of parasite strains between individuals contributes to the distribution and frequency of disease types. Lack of clustering would suggest that alternate risk factors predominate. Thus we evaluated whether ATL, CL, ML and DL occur as clusters of cases in time and space. Our method was to evaluate whether there was a correlation between the frequency of close recent cases of the same clinical disease type and the distances from a newly diagnosed case of CL, DL or ML. Recent cases were defined as ATL patients diagnosed in the three, six or twelve months preceding the diagnosis of the new case.

In these analyses the closest cases varied according to the disease form under consideration. When the entire spectrum of ATL was being considered, the newly diagnosed case and the closest recent case could present any form of the disease (i.e. CL, ML or DL). For example, the new case could present CL and the closest recent case of ATL could present CL, ML or DL. When a specific form of ATL was being considered, then both the newly diagnosed case and the closest recent case should present that same form of disease. For example, if the disease in consideration was DL, then the new and closest recent cases should present DL. So, we started by assessing if leishmaniasis cases have a general tendency to cluster in the affected region, analysing the correlation between number of closest recent cases of ATL in general (i.e. CL, ML or DL) and distance to a new case of leishmaniasis (i.e. again, CL, ML or DL). Then, we investigated if any form of the disease would depart from this general trend, testing the same correlation stratified by clinical presentation of ATL, that is: CL, ML and DL. In such analyses, the recent cases and the newly diagnosed cases would need to be of either CL or ML or DL.

**Fig 2:**
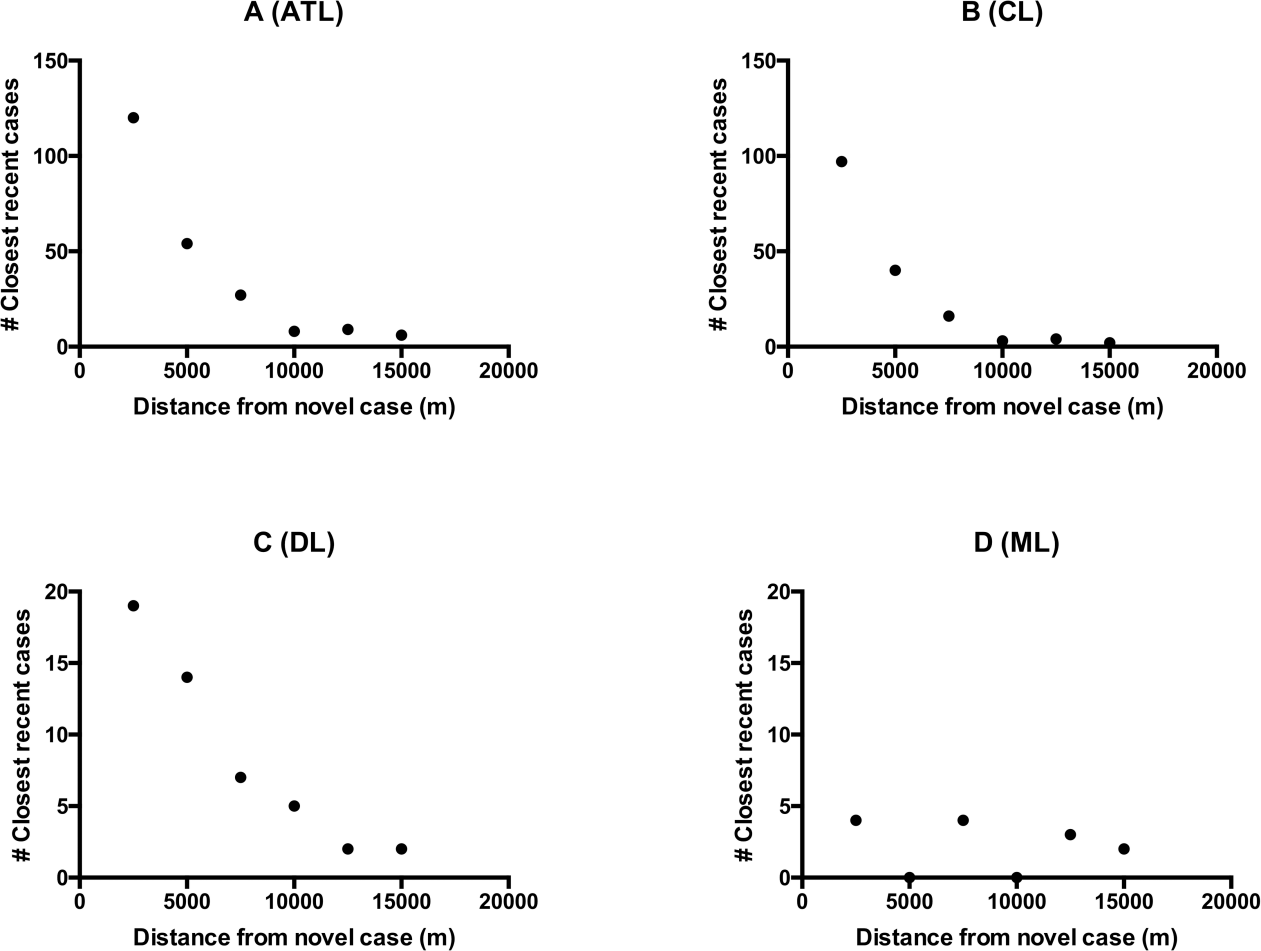
Spearman correlation between frequency of closest recent leishmaniasis cases and distance to a new leishmaniasis diagnosis in Corte de Pedra. Recent cases defined as those cases occurring within the previous six months to a newly diagnosed case are depicted. (A) ATL cases (r = -0.94, p = 0.0083). (B) CL cases (r = -0.94, p = 0.0083). (C) DL cases (r = -0.99, p = 0.0014). (D) ML cases (r = 0.26, p = 0.3292). For each newly diagnosed (i.e. novel) case of leishmaniasis, the closest recent case of the same clinical type was determined. Then, the number of newly diagnosed cases that presented a closest recent case within a discrete 2.5 km distance interval was plotted against the distance interval units. The following distance intervals (in meters) were employed: 0–2500, 2501–5000, 5001–7500, 7501–10000 and 10001–12500.

Considering all forms of ATL together, the number of closest recently diagnosed cases diminished proportionally with the distance from a new case of leishmaniasis (Fig 2A). Statistical correlation analyses confirmed a significant aggregation of ATL patients in all 3 time frames analyzed (Table 2). As an example, within the six months preceding a newly diagnosed case of ATL, approximately 90% of closest recent cases occurred within a maximum 7.5 Km distance from a newly diagnosed patient (ATL within 7.5 Km radius / total ATL: 201/224).

**Table 2:** Spearman coefficient (r) and significance (p) values of the correlations between distances to novel cases and number of novel cases with closest recent cases of ATL, CL, DL or ML at the distance interval units^$^.

ATL form	3 months		6 months		12 months	
	r	p	r	p	r	p
ATL*	-0.99	0.0014	-0.94	0.0083	-0.94	0.0083
CL^%^	-0.94	0.0083	-0.94	0.0083	-0.85	0.0167
DL^#^	-0.81	0.0292	-0.99	0.0014	-0.94	0.0167
ML^&^	0.15	0.4014	-0.26	0.3292	-0.68	0.0681

^$^ For each newly diagnosed (i.e. novel) case of leishmaniasis, the closest recent case of the same clinical type was determined. Then, the number of newly diagnosed cases that presented a closest recent case within a discrete 2.5 km distance interval was plotted against the distance interval units (please, see Fig 2). The following distance intervals (in meters) were employed: 0–2500, 2501–5000, 5001–7500, 7501–10000 and 10001–12500. Recent cases were defined as cases diagnosed in the preceding 3, 6 or 12 months of a newly diagnosed case, depending on the time frame under consideration.

* ATL: American tegumentary leishmaniasis.

^%^ CL: Localized cutaneous leishmaniasis.

^#^ DL: Disseminated leishmaniasis.

^&^ ML: Mucosal leishmaniasis.

Considering the different forms of ATL associated with different strains of *L*. *braziliensis*, we evaluated the aggregation of CL, DL or ML individually within the endemic region. Both CL and DL followed the same pattern of the majority of ATL cases, with approximately 94% (CL within 7.5 Km radius / total CL: 153/162) or 82% (DL within 7.5 Km radius / total DL: 40/49) of closest recent cases occurring within 7.5 km of a new CL or DL case, respectively (Fig 2B and 2C, Table 2). Surprisingly however, there was no correlation between the frequency of recent ML cases and distance to a patient newly diagnosed with ML (Fig 2D, Table 2). Together these data are consistent with clustering of CL and of DL within the 10,000 Km^2^ area of Corte de Pedra. Because CL makes up the majority of all cases, the clustering of all ATL cases reflected the pattern seen in CL. However, ML occurred in a sporadic pattern, suggesting that factors other than the mere spread of index *L*. *braziliensis* strains were influential.

### Trends in ML incidence do not correlate with those of total ATL in the affected area

Besides the distinct pattern of spread over Corte de Pedra, ML also exhibited fluctuation in annual incidence that was distinct from those of CL and DL in the region (Table 3). During the last two study periods, the annual incidence of CL and DL were positively correlated with the total ATL incidence. CL presented correlation coefficients (Pearson r) to ATL of 0.99 (p<0.001) in both study periods, while DL presented correlations of 0.71 (p = 0.037) in 1999–2003, and of 0.90 (p = 0.025) in 2008–2011. No correlation was observed between ML and ATL in either period for which there were data, with the coefficients consisting of -0.15 (p = 0.40) in 1999–2003, and 0.16 (p = 0.42) in 2008–2011.

**Table 3:** Annual incidences of different ATL forms in Corte de Pedra during study years of 1999 to 2003 and 2008 to 2011.

Year	CL^%^	DL^#^	ML^&^	Total ATL*
	n^$^ (%ATL)^!^	n (%ATL)	n (%ATL)	
1999	755 (96.4)	14 (1.8)	14 (1.8)	783
2000	515 (95.5)	8 (1.4)	17 (3.1)	540
2001	625 (93.7)	16 (2.4)	26 (3.9)	667
2002	526 (94.6)	9 (1.6)	21 (3.8)	556
2003	471 (94.8)	8 (1.6)	18 (3.6)	497
2008	910 (94.1)	29 (3.0)	28 (2.9)	967
2009	1122 (93.5)	40 (3.3)	38 (3.2)	1200
2010	1488 (95.6)	46 (3.0)	22 (1.4)	1556
2011	896 (96.0)	24 (2.6)	13 (1.4)	933

* ATL: American tegumentary leishmaniasis.

^%^ CL: Localized cutaneous leishmaniasis.

^#^ DL: Disseminated leishmaniasis.

^&^ ML: Mucosal leishmaniasis.

^$^ n: Number of individuals with that form of ATL that were diagnosed in Corte de Pedra during the year under consideration.

^!^ (%ATL): Proportion of individuals with that form of disease in relation to the total number of ATL patients that was diagnosed in Corte de Pedra during the year under consideration.

## Discussion

Human disease by *Leishmania* spp. of the sub-genus *L*. *Viannia* differs from that due to the *L*. *Leishmania* sub-genus, in that the former more often leads to disseminated and severe forms of tegumentary leishmaniasis. Our prior studies revealed that the parasites causing the different clinical forms of *L*. *braziliensis* disease are genetically distinct, and can be distinguished with a limited number of anonymous markers [13, 15, 16]. During the current study we performed a comparison of the distribution of CL, ML and DL secondary to *L*. *braziliensis* infection at intervals since 1992. The data revealed preferential spread of both CL and DL to geographically closer individuals, but spread of ML occurred independent of proximity to a recent case. This indicates that CL and DL occur in clusters, whereas ML appears in a sporadic pattern within the endemic focus.

We had previously reported the incidence of different forms of ATL in Corte de Pedra during early 2000's [12]. Although in the past ML was the most common disseminated form of leishmaniasis due to *L*. *braziliensis*, DL has emerged and is now more common than ML in that region [6, 7, 17]. Our analyses revealed that DL was distributed over a significantly wider area than ML in 1999–2003 [12]. In the current study, it became apparent that both disseminated forms of ATL were indeed spreading, but in different patterns. One plausible explanation for the clustering patterns of DL and CL propagation would be the occurrence of localized outbreaks of cases due to transmission of DL-prone or CL-prone parasite strains [13, 15].

Reservoir investigation was out of the scope of the current study, but it needs to be carried out to elucidate the mechanism underlying such clustering of CL and DL cases. Possible reasons for the observed clustering might involve the existence of peri-domestic reservoirs in the region, or even a human-vector-human amplification of transmission cycles initiated by peri-domestic or sylvatic hosts and reservoirs.

The observation that ML does not occur in clusters of ill individuals may relate to the fact that it tends to be a late outcome among individuals with past history of CL, and chronically infected with the parasite [5, 18]. For individuals being inoculated with a ML-prone parasite strain, ML might develop only as a complication of CL when certain host or environmental conditions are present [19, 20]. Furthermore, variability in manifestations of this chronic infection might result in different times of mucosal lesions onset among patients, what would interfere with the definition of a cluster, implying temporal and spatial aggregation of cases. For example, some individuals that acquired infection at about the same time might develop symptoms months to years apart, precluding time aggregation. Alternatively, patients might have moved from their original locations, thus masking spatial aggregation. Finally, there may be variability in the time patients seek medical attention upon appearance of symptoms. Thus some individuals might seek treatment before a diagnosis of ML becomes apparent. Although early diagnosis would decrease the incidence of ML, it would not affect CL or DL.

We raise two possible explanations for the observation that ML presented a slower pace of propagation in Corte de Pedra than CL and DL. One plausible explanation would be the longer incubation time of ML. This would cause diagnosis of infections leading to ML to be performed months to years later than diagnosis of those infections that occurred in the population during the same *L*. *braziliensis* transmission season but leaded to CL or DL. The other possibility would be a more frequent and early treatment of all CL cases in this area, where there is naturally more intensive surveillance for and people’s knowledge about ATL. Treatment delay and failure are risk factors for ML development [19, 20]. The early treatment and frequent retreatments might not only prevent part of the ML burden, but also delay its onset and therefore cause the geographic distribution of such cases in this region to be more stationary.

The approach employed in the current study is adequate for the description we provide based on the cross-sectional definitions of cases we used: ATL, CL, DL and ML. However, this approach is inadequate to assess whether individuals that will develop ML in the long run of infection geographically cluster during the initial moments of disease. For addressing this question, the current approach would need to be coupled to a very large cohort of newly diagnosed CL cases. This CL patients cohort would also have to be followed up for several years, in order to detect those few individuals that would develop ML. Only then, the original residence sites of these subjects, where they lived when initially diagnosed with CL, would be retrospectively identified and georeferenced. With these retrospective geographic coordinates, the correlation between frequency of closest recent cases and distances to novel cases of CL patients who would develop ML could be appropriately performed.

Leishmaniases are disorders of the poverty [2], ranking among the world’s most neglected diseases [21]. Control of ATL has been based on treatment of ill individuals [1]; self-protection among dwellers of endemic foci by the use of repellents, proper clothing and bed nets [22–24]; and vector control with insecticides [25]. There is no available safe vaccine or other safe form of prophylaxis for ATL [26, 27]. Likely reasons for the inadequate control of the leishmaniases are: (1) case finding is usually based on self-referral to health care units, and as a result only a fraction of cases are treated [28]; (2) protective measures are not uniformly practiced by the people at risk [29, 30]; and (3) vector control is not systematically employed by the local health agencies [30, 31].

Active surveillance for non-diagnosed cases of ATL could help control disease. However, endemic regions usually span large areas with precarious road systems, precluding comprehensive surveillance measures. As an example, Corte de Pedra spans approximately 8 to 10 thousand square kilometers served by sparse non-paved roads, and hosts a population of about 240,000 inhabitants [17]. The observation that ATL cases cluster in time and space might provide a basis for a targeted approach to active surveillance. This would reduce the area to be covered. Furthermore, even though ML did not show time-space aggregation, its control might benefit from active surveillance to decrease the incidence of CL. This is because ML is often a consequence of late or incompletely treated localized cutaneous leishmaniasis [5, 19].

ATL has been mostly uncontrolled and continues to increase in incidence in large parts of South America [3, 32, 33], despite extensive studies of its epidemiology, geographic features, vector populations [2, 34], and risk factors [19, 30, 35–41]. In addition to existing proposals to prevent human infection with the *Leishmania* spp. [42, 43], a detailed comprehension of the dynamics and propagation of ATL within affected foci will shed fresh light on the factors maintaining leishmaniasis in endemic regions. This could be used to tailor control measures toward early detection of sentinel patients coupled with focused active surveillance for new cases in areas of likely spread. Such surveillance strategy would be aimed at capturing undiagnosed cases, hence decreasing the overall incidence of leishmaniasis in endemic regions.
